# The Effects of Nettle Extract Consumption on Liver PPARs, SIRT1, ACOX1 and Blood Lipid Levels in Male and Female C57Bl6 Mice

**DOI:** 10.3390/nu14214469

**Published:** 2022-10-25

**Authors:** Sandra Domjanić Drozdek, Dyana Odeh, Domagoj Đikić, Romana Gračan, Nada Oršolić, Verica Dragović-Uzelac, Lana Feher-Turković, Petar Dragičević, Irena Landeka Jurčević

**Affiliations:** 1 School of Applied Health Sciences, University of Zagreb, 10000 Zagreb, Croatia; 2 Faculty of Science, University of Zagreb, 10000 Zagreb, Croatia; 3 Faculty of Food Technology and Biotechnology, University of Zagreb, 10000 Zagreb, Croatia; 4 School of Medicine, University of Zagreb, 10000 Zagreb, Croatia

**Keywords:** cholesterol, dyslipidaemia, lipid regulating transcription factors, oxidative stress

## Abstract

The aim of this study was to evaluate how nettle (*Urtica dioica* L.) water extract consumption would interact with regulators of peroxysomal lipid oxidation, histone deacetylase, and markers of oxidative stress in the liver and blood lipid levels in male and female C57Bl6 mice. Metabolically unchallenged (healthy) mice (*n* = 5 per sex) were treated with a nettle extract in a dose of 40 mg of total polyphenols in the extract per kg mice body weight. The nettle extract was applied daily along with normal diet for 15 days. The serum triglycerides, cholesterol, HDL, LDL, and liver PPAR-α, PPAR-γ, PGC-1-α, ACOX1, SIRT1, MDA, SOD, CAT, and GSH were compared between exposed and unexposed (control) animals. In males, the PPAR-α, PGC1-α, and ACOX1 levels together with systemic HDL cholesterol were significantly (*p* ≤ 0.05) increased while the LDL cholesterol decreased (*p* ≤ 0.05). In females, no changes in PPAR-α and PGC1-α or serum lipids were noted, but the ACOX1 content in the liver was significantly (*p* ≤ 0.05) increased. The SIRT1 activity increased (*p* ≤ 0.05) only in females. In both sexes, the PPAR-γ levels were not significantly (*p* ≤ 0.05) affected in either sex. The results indicate that nettle plant extract has the potential to modulate selected transcriptional factors and histone deacetylase in vivo, with certain sex differences, which should be studied further in similar models.

## 1. Introduction

The stinging nettle (*Urtica dioica* L.) is a subject of extensive in vitro/in vivo and clinical studies. It is one of the most promising plants used as herbal supplement with positive effects on hypertension, diabetes, obesity, and metabolic syndrome [1,2,3]. The beneficial properties following plant consumption or extract intake are extensively evaluated scientifically for molecular, biochemical, and physiological mechanisms through various evidence-based scientific experiments [1,2,3].

In the most comprehensive review by Samakar et al. [1], it is presented how nettle consumption exhibits various positive effects in metabolic conditions. In hypertension, the hypotensive features of nettle consumption are achieved through various pathways such as vasorelaxation [1,4,5,6,7]. The regulation of glucose in diabetes and the antidiabetic features of nettle extract on lowering blood glucose are achieved through the activation of pancreatic or non-pancreatic pathways [1,8,9,10,11,12,13]. The lipid metabolism modulating effects are reflected through the potential of nettle extracts to elevate the levels of high-density lipoprotein (HDL) particles and lower the low-density lipoprotein (LDL) particle levels to prevent atherogenic dyslipidemia [1]. Atherogenic dyslipidemia is characterized by increased blood concentrations of LDL, decreased HDL, and increased total triglycerides and cholesterol [14,15]. Nettle consumption causes metabolic balancing in atherogenc dyslipidemia and hyperlipidemia by boosting lipid metabolism, increasing the oxidation of fatty acids, stabilizing lipid peroxidation, and reducing oxidative stress in the liver [1,14,16,17,18].

Nettle has an array of biologically active molecules responsible for such physiological modulating properties [19,20]. The upper plant parts are rich in terpenoids, carotenoids, fatty acids, essential amino acids, chlorophylls, vitamins, carbohydrates, nitrates, sterols, polysaccharides, isolectins, minerals, and polyphenols, for example, quercetin as the most examined one, or rutin and sinapic acid as the most predominant ones, followed by gallic, caffeic, chlorogenic and p-coumaric acid, naringenin, naringin, cinnamic acids, and flavonols [19,20,21]. Nettle’s essential oil contains at least 43 compounds, the main ones being carvacrol, carvone, naphthalene, anethol, hexahydrofarnesyl acetone, geranyl acetone, β-ionone, and phytol, among others [1].

Nettle extract consumption and its bioactive molecule interaction with metabolic regulating proteins, transcription factors, secondary messenger hormones, etc., is being elucidated through experimental work. For example, a well-known effects on adipokine apelin and C-reactive protein (hs-CRP) plasma levels [22], as well as an increase in K-Ras and decrease in glycogen synthase kinase-3 beta [23], have been described. 

Among regulatory molecules, the peroxisome proliferator activated receptors (PPARs) are nuclear receptors that play a crucial role in regulating lipid and glucose homeostasis. Being activated by free fatty acids as endogenous ligands, the three PPAR subtypes -α, -γ, and -δ (also referred to as β) function as lipid sensors in animal and human organisms [24]. When activated, PPARs mainly act as transcription factors that bind with the respective response elements on the DNA and enhance the expression of genes of primary metabolism, leading to the production of, e.g., lipoproteinlipase (LPL) or membranous glucose transporters (GLUT), lipid oxidizing enzymes (in peroxisomes and mitochondria) or gluconeogenesis enzymes, etc. [24]. In this way, the cellular adaptation of energy consumption to the nutrient supply is achieved [24]. The consumption of medicinal plants such as nettle, along with or instead of currently available chemical drugs, could perhaps improve the management of hypertriglyceridemic patients, since herbal products are an important part of the treatment of dyslipidemia due to their popularity, availability, price, and safety [2,24]. 

Interestingly, the amount of experimental work on interactions of PPARs and lipid-oxidizing enzymes and nettle is rather scarce, although some evidence exists, for example, the earliest work by Rau et al. [24] in cell culture, or more recent work by Fan et al. [25] on the gene expression mRNA level in rat tissues in vivo, which accentuated that, in addition to changes in mRNA expression, further studies on protein expression levels in vivo are necessary [25].

Therefore, the aim of our study was to evaluate how natural polyphenol-rich nettle-water-extract consumption would interact with the levels or activity of major transcription factors, the peroxisome-proliferator-activated receptors (PPARs, subtypes -α, -γ) and peroxisome-proliferator activated receptor gamma coactivator 1 α (PGC1-α cofactor), histone deacetylase sirtuin 1 (SIRT1), lipid-oxidizing enzyme in peroxisomes acyl CoA oxidase 1 (ACOX1), markers of oxidative damage malondialdehyde (MDA), carbonylated proteins, superoxide dismutase (SOD), catalase (CAT), reduced glutathione (GSH) in the liver and systemic blood lipid levels in vivo in the metabolically unchallenged (healthy) C57Bl6 mice of both sexes.

## 2. Methods and Materials

### 2.1. Experimental Animals and Treatment and Experimental Design

A total of 20 inbred C57Bl6 mice were included in this experiment, the males (*n* = 10) weighing 30 ± 1.5 g and females weighing 25 ± 2.0 g (*n* = 10), obtained from the University of Zagreb Faculty of Science Department of Animal Physiology. Animals had free access to a standard laboratory diet (4 RF 21, Mucedola Srl, Settimo Milanese, Italy) and tap water and were kept under a 12/12 h light cycle under standard housing conditions for laboratory mice described by the international guidelines [26], and the experiments were approved by the Bioethics Committee of the Zagreb University Faculty of Science (Approval No: 251-58-10617-17-7) and State Bioethical cometee of the Republic of Croatia (Approval No: UP/I-322-01/21-01/01) and EU Directive 2010/63/EU.

Animals were randomly divided into four groups: control males (*n* = 5), control females (*n* = 5), nettle extract males (*n* = 5), and nettle extract females (*n* = 5). The males and females in the nettle extract group that received the dose of nettle extract were given as a concentration of total polyphenols of 40 mg per kg of body weight of the mouse in the volume of 0.2 mL per animal. The preparation of the nettle extract that was given to the mice, together with the composition and concentrations of bioactive phenolic molecules, is described in the previously published work of Elez Garofulic et al. [27]. The nettle extract that showed the highest polyphenol content of individual bioactive polyphenols (the extract designated under the name PLE in the work of Elez-Garofulic et al. [27]) was used in this experiment. The rationale for the depiction of the dose given daily to mice was estimated by comparing the doses listed in the review of Samakar et al. [1], adjusted to the body weight of mice by standard pharmacological allometric scaling for animal dosing [28]. The PBS (control) or nettle extract were administered daily as maximum of 0.2 mL water solution by gavage for 15 days between 8 and 10 a.m. to avoid differences in metabolism related to the circadian rhythm. After 15 days of treatment, 24 h after the last treatment, the animals were anaesthetized with isoflurane (3% in O_2_ flow 0.8–1.5 L/min) and injected with a mix of 100 mg/kg ketamine and 10 mg/kg xylazine for blood and liver tissue collection, after which the animals were sacrificed by cervical dislocation.

### 2.2. Blood and Liver Tissue Preparation

Blood was collected by cardiac puncture and centrifuged to isolate serum for analysis of lipid parameters. The serum was immediately frozen at −80 °C until analysis. Livers were isolated and parts of tissue samples were placed in 50 mmol/L phosphate-buffered saline (PBS, pH = 7.4) and homogenized (10% of homogenate by tissue mass per volume of PBS) with an ultrasonic homogenizer (SONOPLUS HD2070, Bandelin Electronic GmbH & Co KG, Hagen, Germany) with an MS73 probe (Bandelin, Electronic, Hagen, Germany). Sonication of liver homogenates was performed on ice for 30 s in three 10 s intervals. Homogenates were centrifuged at 4 °C and 20,000× *g* for 15 min, and supernatants were separated and immediately frozen at −80 °C until the analysis of PPARs, ACOX1, and oxidative stress parameters.

### 2.3. Liver PPAR-α, PPAR-γ, PGC1-α, ACOX1, SIRT1 Assays

The levels of peroxisome proliferator-activated receptor -α and -γ (PPAR-α, PPAR-γ), peroxisome proliferator-activated receptor -γ coactivator 1--α (PPARGC1A, i.e., PGC1--α) levels and acyl-coenzyme A oxidase 1 (ACOX1) concentration in mouse liver tissue homogenates we used corresponding My BioSource microwell strip plate enzyme-linked immunosorbent assay (ELISA) kits (Catalogue Nos. MBS2502542, MBS701124, MBS707053, and MBS9316691 respectively), and for the SIRT1 activity in mouse liver, we used an Abcam 156915 Universal SIRT Activity Assay Kit (Colorimetric) according to the manufacturer’s instructions. The color intensity for all three kits was measured on a Tecan Infinite 200 microplate reader.

### 2.4. Serum Lipids and Serum Biochemistry

Lipids were analysed with the Beckman Coulter OSR61118 kit, total cholesterol with the OSR6216 kit, and HDL cholesterol with OSR6195 (Beckman Coulter, Brea, CA, USA) according to the manufacturer’s instructions. Chromophore reactions yield a blue color complex, which can be measured bichromatically at 650 nm with OSR61118 and OSR6195 kits, and OSR6216 kit reactions yielded red quinoneimine dye, measured spectrophotometrically at 540/600 nm as an increase in absorbance, on an Infinite 200 plate reader (Tecan company, Hamburg, Germany); each kit had a coefficient of variation of up to 5%. LDL was estimated from measured parameters by the method described in Friedwald et al. [29] and Oršolić et al. [30].

### 2.5. Liver Oxidative Stress Analysis

To analyze markers of oxidative stress as described below, the frozen tissue supernatants as described above were thawed on +6 °C and then centrifuged at 4 °C and 20,000× *g* for 15 min and used.

The protein concentrations in liver tissue homogenates were used to express all biochemical and molecular parameters as units per mg of protein, determined according to Lowry et al. [31]. Bovine serum albumin (BSA, Sigma Aldrich, Darmstadt, Germany) was used as the standard.

Malondialdehyde (MDA), a marker of lipid peroxidation, was determined by a modified method described previously [32]. Briefly, a 200 µL sample of homogenized liver supernatant was mixed with 200 µL of 8.1% sodium dodecyl sulphate (SDS), 1.5 mL of 20% acetic acid (pH = 3.5), and 1.5 mL of 0.81% thiobarbituric acid and incubated at 95 °C for 60 min. After rapid cooling on ice, the absorbance was measured at 532 and 600 nm with a Libro S22 ultraviolet–visible (UV–Vis) spectrophotometer (Biochrom Ltd. Cambridge, UK). The total absorbance was determined by deducting absorbance at 600 nm from the absorbance measured at λ = 532 nm. The MDA levels were then determined using the molar extinction coefficient for malondialdehyde-thiobarbiturate (MDA–TBA) complex of 1.56 × 10^5^ and expressed as nmol/mg of protein in tissue homogenate.

Carbonylated proteins in the liver homogenates were determined by adding 200 µL of sample homogenate to 300 µL of 10 mmol/L 2,4-dinitrophenylhydrazine (DNPH) in 2 mol/L HCl and shaking for 1 h at room temperature. Proteins were precipitated with 10% (*w*/*v*) trichloroacetic acid (TCA) at −20 °C for 5 min and then centrifuged at 4 °C and 12,000× *g* for 10 min. The supernatant was removed and the precipitate resuspended in a 1:1 mixture of ethanol and ethyl acetate and centrifuged under the same conditions. The obtained pellet was washed repeatedly five times until all unbound DNPH was removed. The residue was then dissolved in 6 mol/L guanidine HCl at 35 °C. In the resulting solution, the carbonylated protein concentration was measured at an absorbance of λ = 370 nm and calculated by using the molar extinction coefficient 22,000 M^−1^cm^−1^ and expressed as nmol/mg protein [33].

The superoxide dismutase (SOD) activity was determined as described previously [32]. The tissue homogenate (25 µL) was mixed with 1.45 mL of reaction solution (0.05 mmol/L of cytochrome C and 1 mmol/L of xanthin mixed with 5,5’-dithiobis(2-nitrobenzoic acid) (DTNB) in a 10:1 (*v*/*v*) ratio). The 20 µL of xantine oxidase (0.4 U/mL) was added to start the reaction. The absorbance was measured with the UV–Vis spectrophotometer mentioned above at λ = 550 nm for 3 min. One unit of SOD activity was defined as the amount of enzyme required to cause 50% of the inhibition of superoxide anion production within the sample. The results were expressed as units per mg of protein in tissue homogenate (U/mg protein).

The liver catalase (CAT) activity was assayed by measuring the initial rate of hydrogen peroxide degradation [32]. The reaction mixture was prepared by mixing 33 mmol/L H_2_O_2_ in 50 mmol/L phosphate buffer (pH = 7.0). Next, 900 µL of the prepared reaction mixture was mixed with 100 µL of tissue supernatant, and the absorbance was measured with a UV–Vis spectrophotometer at λ = λ = 240 nm for 3 min. The CAT activity was calculated using the molar extinction coefficient of 43.6 L/mol cm for H_2_O_2_. The results were expressed as U/mg protein.

Reduced glutathione (GSH) determination was described earlier [32]. Briefly, 40 µL of 10 mmol/L DTNB was added to each well of a 96-well plate containing 20 µL of tissue supernatant (obtained as described above) pre-treated with 40 µL of 350 mmol/L HCL and incubated for 10 min. Then, 100 µL of reaction solution was prepared earlier by mixing 9980 µL of 0.8 mmol/L nicotinamide adenine dinucleotide phosphate (NADPH) and 20 µL of 0.2 U/Ml glutathione reductase and analysed at λ = 412 nm for 5 min in an ELISA plate reader (Biorad Laboratories, Hercules, CA, USA). The GSH levels were determined from the calibration curve of GSH standards. The results are expressed as µmol/mg protein.

### 2.6. Body Weight and Relative Mass of Liver (the Liver Organosomatic Index)

Animal body weight was monitored by weighing the animals on the first and last day of the experiment using a digital scale (Kern KB 2000-2N, Balingen, Germany; d = 0.01–2000 g). The body-weight change was a difference in mass between the last and first experimental day, as presented in the results section.

At the end of the experiment, the livers were isolated and weighed on a digital scale (Electronic balance ABS 220-4, Kern & Sohn, Balingen, Germany).

The effect of nettle extract consumption on the relative mass of liver was calculated according to the formula:

Liver organosomatic index (%_body weight_) = (final organ mass/final rat mass) × 100.

### 2.7. Serum Liver Markers

The unhemolyzed serum was collected and frozen at −80 °C until the further processing of biochemical parameters. All biochemistry analyses were conducted according to the recommendations of the International Federation of Clinical Chemistry (IFCC) methods in enzymology and were performed with commercial kits (Sigma-Aldrich, St. Louis, MO, USA) on the Hitachi 717 automatic analyzer (Hitachi, Tokyo, Japan). The serum biochemical parameters of hepatic function included the concentration of aspartate aminotransferase (AST-U/L), alanine aminotransferase (ALT-U/L) and lactate dehydrogenase (LDH-U/L).

### 2.8. Statistical Analysis

All the data are expressed as means ± standard deviations (SD). GraphPad Prism 9 (GraphPad Software, San Diego, CA, USA) [34] was used for data analysis and statistical comparisons between the groups with Kruskal–Wallis and Tukey’s test. The level of significance was set to *p* < 0.05.

## 3. Results

### 3.1. The Effects of Nettle Extract Consumption on Transcription Factors Regulators (PPARs), ACOX1 and SIRT1

**Figure 1:**
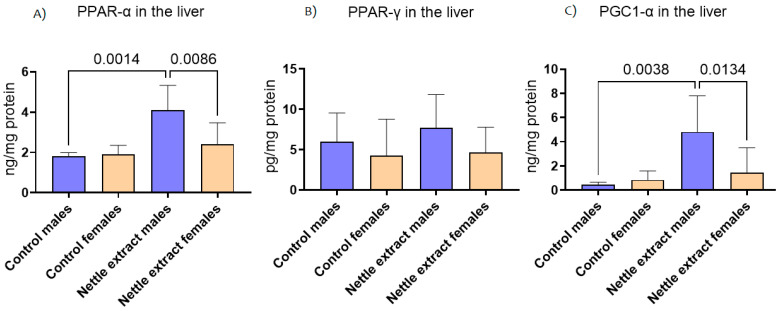
The changes in PPAR-α, PPAR-γ and PGC1-α in the liver of C57Bl6 mice treated with nettle extract. The numbers above bars are *p* values meaning statistically significant (*p* ≤ 0.05) differences between the treated groups by Kruskal–Wallis ANOVA.

The PPAR-α level was significantly (*p* ≤ 0.05) increased after 15 days of nettle extract consumption in male animals (Figure 1). In females, nettle consumption did not significantly (*p* ≤ 0.05) affect the PPAR-α level (Figure 1). In both sexes, the PPAR-γ levels were not significantly (*p* ≤ 0.05) affected by nettle extract consumption (Figure 1). The PGC1-α level was significantly (*p* ≤ 0.05) increased after 15 days of nettle extract consumption in male animals (Figure 1). In females, nettle consumption did not significantly (*p* ≤ 0.05) affect the PGC1-α level (Figure 1).

**Figure 2:**
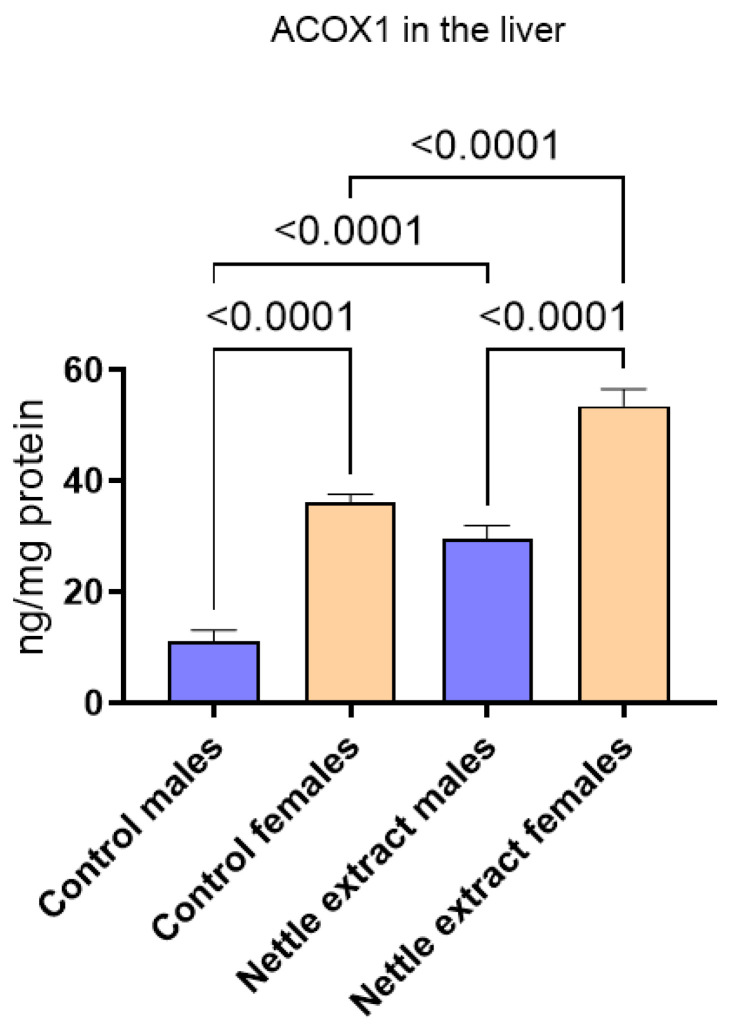
The changes in PGC1-α in the liver of C57Bl6 mice treated with nettle extract. The numbers above bars are *p* values meaning statistically significant (*p* ≤ 0.05) differences between the treated groups by Kruskal–Wallis ANOVA.

After nettle extract consumption, the ACOX1 level was significantly (*p* ≤ 0.05) elevated in both males and females (Figure 2).

**Figure 3:**
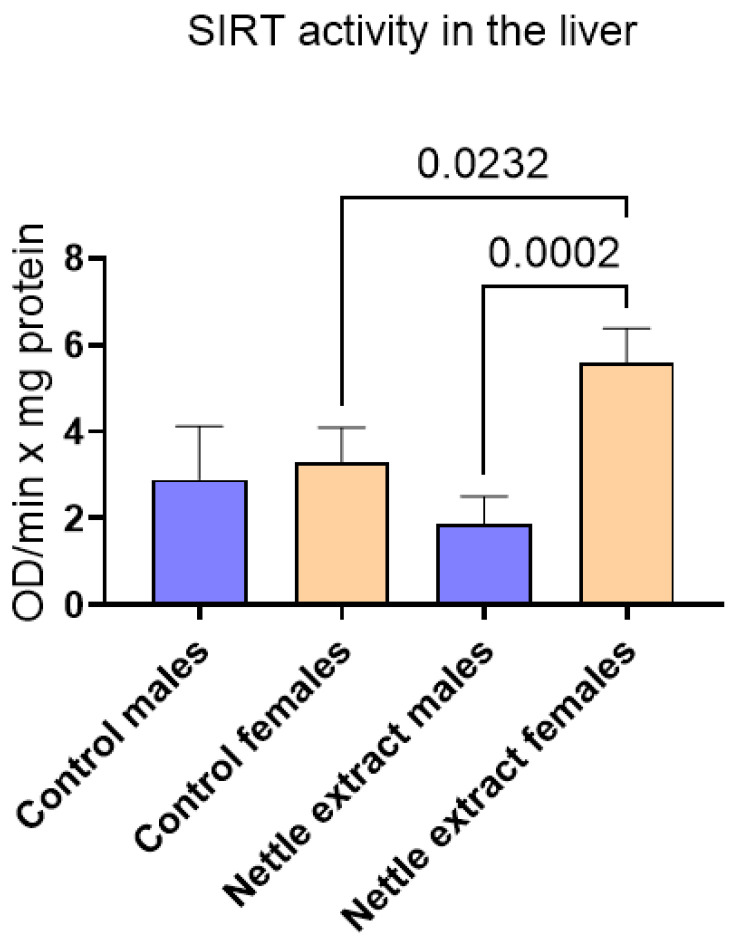
The changes in SIRT1 activity in the liver of C57Bl6 mice treated with nettle extract. The numbers above bars are *p* values meaning statistically significant (*p* ≤ 0.05) differences between the treated groups by Kruskal–Wallis ANOVA.

The SIRT1 level was significantly (*p* ≤ 0.05) elevated in females but remained unchanged in males after nettle extract consumption (Figure 3).

### 3.2. The Effects of Nettle Extract Consumption on Blood Lipid Levels (Triglycerides, Total Cholesterol, HDL and LDL) and Blood Biochemistry Parameters

**Figure 4:**
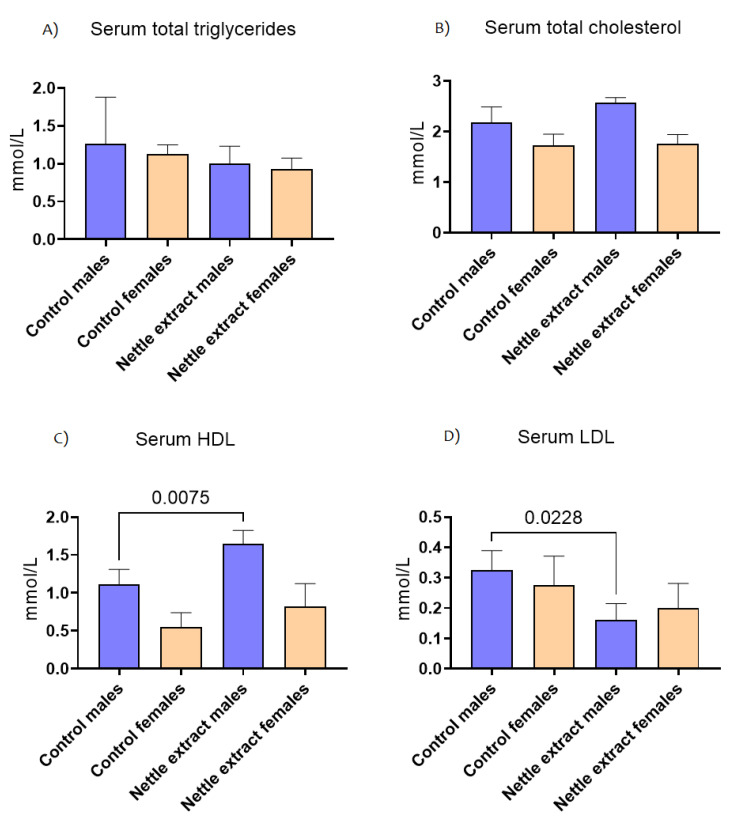
The changes in blood lipid level of C57Bl6 mice treated with nettle extract. The numbers above bars are *p* values meaning statistically significant (*p* ≤ 0.05) differences between the treated groups by Kruskal–Wallis ANOVA.

The concentration of total triglycerides and total cholesterol in the blood did not significantly (*p* ≤ 0.05) change after nettle consumption in both sexes (Figure 4). The HDL cholesterol lipoprotein particles were significantly (*p* ≤ 0.05) increased and LDL cholesterol decreased (*p* ≤ 0.05) in males. In females, no significant change in HDL or LDL cholesterol was observed (Figure 4).

### 3.3. The Effects of Nettle Extract on Major Antioxidative Defense Markers in Liver Tissue

**Figure 5:**
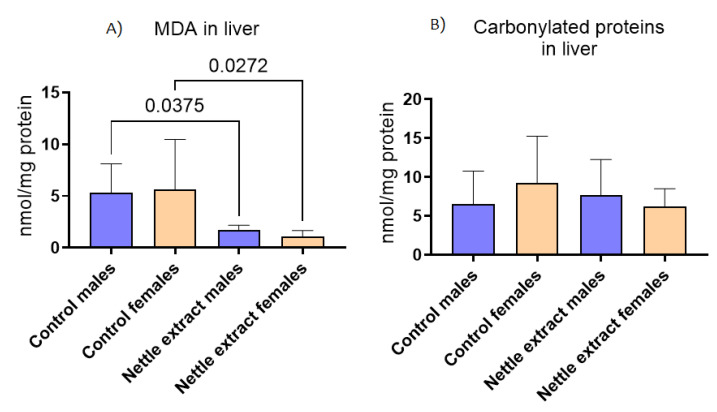
The changes in (**A**) malonaldehyde (MDA) and (**B**) carbonylated proteins in the liver of C57Bl6 mice treated with nettle extract. The numbers above bars are *p* values meaning statistically significant (*p* ≤ 0.05) differences between the treated groups by Kruskal–Wallis ANOVA.

The concentration of malondyaldehide (MDA), a marker of lipid peroxidation, significantly (*p* ≤ 0.05) decreased after nettle consumption in both sexes (Figure 5), while the concentration of carbonylated proteins (Figure 5) was not different in exposed and unexposed animals of both sexes.

The changes in major antioxidative defense enzymes and molecules showed that, in the liver of C57Bl6 mice treated with nettle extract (Table 1), only reduced glutathione (GSH) was significantly (*p* ≤ 0.05) decreased in both sexes.

### 3.4. The Change in Body Mass and Orgonasomatic Index of the Liver

**Figure 6:**
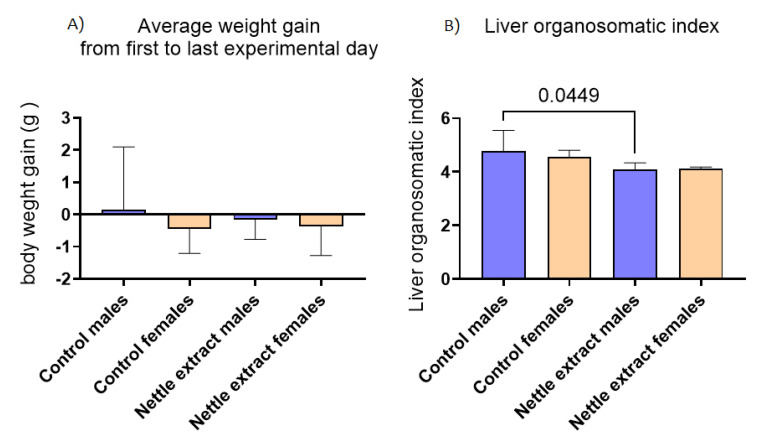
The changes in body weight and liver organosomatic index in the liver of C57Bl6 mice treated with nettle extract. The numbers above bars are *p* values meaning statistically significant (*p* ≤ 0.05) differences between the treated groups by Kruskal–Wallis ANOVA.

In both sexes, the nettle extract consumption did not significantly (*p* ≤ 0.05) affect changes in body weight but significantly (*p* ≤ 0.05) lowered the liver organosmatic index in males (Figure 6).

Changes in blood chemistry liver markers of C57Bl6 mice treated with nettle extract (Table 2) show that only the lactate dehydrogenase (LDH) was significantly (*p* ≤ 0.05) elevated in both sexes.

## 4. Discussion

The results of this experimental model show that nettle extract consumption affected the activation of transcription factors that control lipid metabolism, differently in each sex. It seems, according to the results, that within 15 days and with a specified intake dose, the nettle extract can increase the HDL lipoprotein cholesterol particles (the good cholesterol) significantly (*p* ≤ 0.05) in males but not in female C57BL6 mice. Similar as in this mouse model, in humans, there are reported clinical trials that show a similar HDL increase due to nettle consumption [35,36]. However, human clinical studies also show a general decrease in the cholesterol level in blood and sometimes even decrease in the total serum triglycerides in pathologic states, which were not recorded here. For example, Dadvar et al. [35], in an 8-week consumption study, reported the effect of nettle consumption combined with the aerobic training on the lipid profile in diabetic patients, where the effects of increasing HDL were noted [35]. Another unblinded prospective interventional study included 119 diabetic patients receiving the composition (1 g TDS) but with the addition to their usual medications. After 12 weeks, their lipid condition showed decreased levels of mean total cholesterol and mean serum TG [36].

Similar to clinical studies with patients in the literature, the results from animal experiments exist but also with metabolically challenged animals by alloxan/streptozotocin (STZ) diabetes or high-fat/cholesterol-diet-induced dyslipidemia and obesity experiments. Mehran et al. [37] observed lowered serum TG, cholesterol, LDL, and LDL/HDL ratio in diabetic male Wistar rats induced by STZ for 28 days with the consumption of nettle and *Lamium album* extracts (100 mg/kg/day). A decrease in the serum cholesterol level after treatment with both plant extracts was observed. In addition, the serum LDL, LDL/HDL ratio, and TG level were lowered while the serum HDL level was elevated in rats treated with both plant extracts separately. The decrease in serum TG in rats exposed to nettle was more pronounced than *L. album* extract [37]. Das et al. [38] also used STZ-induced diabetic rats exposed to an aqueous extract of nettle (1.25 g/kg). After 4 weeks, a significant reduction in the level of cholesterol was observed and, similar to our study, the HDL level increased [38]. Other experiments included rats subjected to and challenged by high-fat or high-cholesterol diets. Hypocholesterolemic effects were observed in male rats with a high-cholesterol diet in a 4-week period who received nettle extract (100 or 300 mg/kg) or lovastatin (10 mg/kg) as the control group [39]. The cholesterol, LDL, and LDL/HDL ratio were decreased, which caused the blood lipid profile to improve [39]. The influence of a herbal composition including nettle leaves (100 mg/kg) and burdock (*Arctium lappa*) root extracts (25 mg/kg) on lipid metabolism has been investigated in 90 male diabetic rats in a 10-day trial. As a result, hypertriglyceridemia and lipoperoxidation were effectively decreased [16].

Samakar et al. [1], in his review, summarized a total of seven antihyperlipidaemic studies with an HDL increase in rat models and five studies using rat models [1]. It is important to note that the main difference from the named human or animal studies [1,16,35,36,37,38,39] and our study was that, in our experiment, we used metabolically unchallenged and uncompromised healthy animals. The clinical and animal studies, as a difference from our experimental design, were conducted in metabolically disturbed, diabetic subjects and reported dyslipidaemia reductions occurred in patients or animals with the pathologically high lipid levels that are usually present in metabolic illnesses or accompany diabetes and metabolic syndrome. In diabetic patients and animals, the lipid serum level in metabolically disturbed subjects was up to 50% more elevated than that in control animals [1,16,35,36,37,38,39]. The observed reductions in TG, cholesterol, and LDL and increase in HDL were attributed to improvements (decrease lipids) from such pathological conditions, and the lowered TG, cholesterol, and LDL and raised HDL only approximately approached but were never fully restored to the normal physiological levels [1,16,35,36,37,38,39]. We wanted to determine nettle extract consumption effects on the physiological baseline lipid levels and determine how transcription factors and lipid markers are modified under normal physiological conditions to elucidate the molecular interactions that occur in healthy organisms. The reasoning for this approach was to simulate what happens if generally healthy individuals from the population consume nettle extract as a dietary supplement, which is nowadays readily available. Our results imply that the effects of the consummation of nettle extract could be beneficial in modulating and increasing the HDL and decreasing the LDL cholesterol levels as a prophylaxes supplementation, at least in one of the sexes. The second difference, and to a certain level, the limitation of this study, was that we used a shorter exposure of nettle extract compared to other studies using at least 100 mg/kg (or higher) of nettle extract with a minimum of 30 days of exposure in either humans or animals [1]. In addition, it is important to stress that the interspecies differences in the physiology of mice, rats and humans certainly contribute to differences in observed effects. The slight differences between references and the results presented here and limitations of this study are a reflection of interspecies differences, pathologic/non-pathologic physiology, doses, and a shorter time of exposure. Perhaps, a different regime of exposure (prolonged) with higher doses would affect the female metabolism as well, which remains to be confirmed by further experiments. Furthermore, the lactate dehydrogenase (LDH) increase in blood also draws attention as an additional limitation of the study. LDH is an important enzyme that helps with cellular respiration [40]. A higher-than-normal total LDH means possible tissue damage, usually to the liver, heart, or erythrocytes, depending on the circulating isoform [40]. Additionally, as an essential enzyme for anaerobic respiration, LDH production increases under hypoxic conditions in hepatocytes [40]. Elevated blood LDH ould also be the result of or extensive metabolism and oxygen consumption in the tissues through oxidizing processes (such as lipid oxidation), the renewal of hepatocytes or erythrocytes, and the removal of older cells [40].

In general, the molecular regulators and mechanisms responsible for the observed changes in circulating lipid levels and metabolism could be varied. Previous evidence by Pourahmadi et al. [41] shows that, for example, nettle root extract decreased 3-hydroxy-3-methyl-glutaryl-CoA (HMG-COA) reductase activity, which causes a lowering effect on the total cholesterol and plasma LDL levels in rats [1,41]. However, especially scarce are the references showing the mechanism measuring the effect of nettle on activating the peroxisome proliferator-activated receptors (PPAR); one example is one of the early works by Rau et. al. [24] performed on Cos7 cells transfected with Gal4-driven luciferase reporter plasmid (pFR-Luc/Stratagene) with the ligand binding domain of the respective human PPAR subtype cloned downstream of the Gal4-binding domain. In that experiment [24], among 52 plant extracts, nearly 50% significantly activated PPAR-γ, and only 14 plant extracts activated the PPAR-α [24]. However, only three plant extracts in that work were pan-PPAR activators. Nettle leaf extract was one of those three [24]. Recent work by Fan et al. [25] in a 12-week dietary intervention study on the effect of nettle as a food supplement on high-fat-diet-induced obesity and insulin resistance in C57BL/6 showed that PPAR-α mice showed fasting-induced transcription (mRNA levels). An increase in transcription was observed in the adipocyte factor (FIAF) in adipose and skeletal muscle, the peroxisome-proliferator-activated receptor-α (PPAR-α) and forkhead box protein (FOXO1), and carnitine palmitoyltransferase (Cpt1) in muscle and liver [25]. Fan et al. [25] emphasized that the assessed mRNA changes (transcript abundance) have yet to be quantified and confirmed through changes in the protein levels encoded by these, which we tried to achieve in our work. Furthermore, Chatterji et al. [36] showed the upregulation of PPAR -α after nettle extract consumption and an increase in the oxidation of fatty acids in the liver, which is associated with large reductions in serum lipids and adipose tissue mass [36]. Such regulation of lipids and reported upregulation of PPAR–α [24,25,36] corroborate with the effects observed in male animals in our work. We think, based on the results in males, that PPAR-α transcriptional pathways are major pathways that govern the upregulation of ACOX1 lipid-oxidizing enzyme in peroxisomes, which affected the removal of long-chain fatty acids through that process, resulting in lowered LDL/increased HDL lipoprotein particles in blood. The PPAR-α, mainly being expressed in the liver, leads to an increase in hepatic fatty acid uptake from circulating lipoprotein, resulting in lower serum levels of LDL. This effect is clinically used with lipid-lowering drugs, such as synthetic activators of PPAR-α (fibrates) and PPAR-γ (glitazones) [24]. PPAR-α and PGC1-α were significantly (*p* ≤ 0.05) increased after 15 days of nettle extract consumption in male animals, implying an increase in the control of fat-oxidizing enzyme genes. Usually, as a main biological function, the PPAR-α is regarded to affect the increase in the fatty acid uptake and fat intracellular binding, mitochondrial β-oxidation and peroxisomal fatty acid oxidation (through ACOX1), ketogenesis, triglyceride turnover, gluconeogenesis, and bile synthesis/secret. Since ACOX1 was significantly (*p* ≤ 0.05) elevated, it could be speculated that the peroxisomal oxidation of fat in the male liver was reinforced [42,43]. Endogenous ligands, for example, for PPAR-α, include fatty acids such as arachidonic acid, other polyunsaturated fatty acids or some fatty acid-derived compounds such as arachidonic acid metabolites, the 15-hydroxyeicosatetraenoic acid family, 15(S)-HETE, 15(R)-HETE, and 15(S)-HpETE and 13-hydroxyoctadecadienoic acid, a linoleic acid metabolite. Synthetic ligands include the fibrate drugs, which are used to treat hyperlipidemia, and a diverse set of insecticides, herbicides, plasticizers, and organic solvents collectively referred to as peroxisome proliferators [42,43]. Fibrates effectively lower serum triglycerides and raise the serum HDL cholesterol levels [18] regulated by free fatty acids, and they are a major regulator of lipid metabolism in the liver [42,43]. Since nettle extract contains at least 30 phenolic water-soluble and around 40 volatile/oil biomolecules named in the introduction [1,19,20], some of them certainly can act as ligands or activators of the regulatory proteins examined here. The experiments with each separate biomolecule are necessary to confirm its ligand and activating properties on PPRs and sirtuins.

In either males or females, the PPAR-γ levels were not significantly (*p* ≤ 0.05) affected. PPAR-γ is usually regarded as a transcription factor that controls the storage of lipids in adipocytes, by controlling an array of genes that produce enzymes and protein molecules involved in the control of fat deposit increase. PPAR-γ increases the insulin sensitivity by enhancing the storage of fatty acids in fat cells (reducing lipotoxicity) and by enhancing adiponectin release from fat cells [44]. Low PPAR-γ reduces the capacity of adipose tissue to store fat, resulting in increased storage of fat in nonadipose tissue (lipotoxicity) [44]. PPAR-γ knockout mice are devoid of adipose tissue, establishing PPAR-γ as a master regulator of adipocyte differentiation [44]. According to the results of this experiment, regarding the unchanged PPAR-γ levels in males, it can be speculated that, in males, the nettle consumption did not disrupt the fat storage rate in adipocytes or influence the rate of gluconeogenesis and insulin sensitivity. The results of the unchanged total triglyceride levels in serum support such conclusion.

However, in females, in contrast to males, the nettle extract consumption did not significantly (*p* ≤ 0.05) affect the PPAR-α, PGC1-α, or PPAR-γ levels. Nevertheless, the ACOX1 was significantly (*p* ≤ 0.05) elevated in females. Thus, it seems that, in females, different than in males, the PPAR-α transcriptional pathway is not the major pathway that governs the upregulation of ACOX1 lipid-oxidizing enzyme in peroxisomes. On an epigenetic level, the SIRT1 histone deacetylase activity was increased in bot sex. SIRT1 is a well-known key player and stress-dependent metabolic sensor that can deacetylate many downstream key transcriptional factors in both metabolism-dependent and -independent metabolic pathways to affect metabolism or mitochondrial function and biogenesis; other than PPARs, activated proteins by SIRT1 in the liver include forkhead box protein O1 (AKT/FOXOpathway), uncoupling proteins (UCP), liver X receptor (LXR), p65 protein, mTOR that activates SREBP1/2, and others [45,46]. SIRT1 is usually activated by the NAD/NADH ratio in cells [45]. Consequently, at this point, we can only hypothesize that some of the named pathways activated by SIRT1 led to the activation of ACOX1 in females, but without further experiments, such assumptions remain to be studied further. There is also a possibility that the nettle extract consumption differences occurred due to the physiological differences in males or in females, which include hormones and the hormonal enzymatic pathways of biotransformation enzymes that differently metabolize the bioactive molecules in nettle extract [1,19,20]. Perhaps some of the cytochrome P450 (CYP) enzymes, especially female aromatase (CYP19), a cytochrome P450 enzyme that catalyzes a critical step in the conversion of androgens (C19 steroids) to estrogens (C18 steroids) and is involved in biotransformation of many xenobiotics and farmaceuticals, degraded some of bioactive components of nettle extract [47]. Such biotransformation and possible degradation of bioactive molecules, which were active in males, diminished its effects in females. Further experiments should test such assumptions.

## 5. Conclusions

The nettle extract consumption can increase the level of liver peroxysomal acyl oxidase 1 (ACOX1). On the level of transcriptional factors in the liver, the increase in PPAR-α, which is usually connected as one of the controlling factors for the transcription of ACOX1 and other lipid-metabolizing enzymes and proteins, is sex dependent. The applied dose and time of exposure was enough to increase the level of this transcriptional factor in males and not in females. However, on the epigenetic level of SIRT1, the females show an increase in this protein. The differentially activated molecules act as different ligands in both sexes and perhaps boost different pathways, leading to ACOX1 activation, and such differences occur on a systemic level among circulating blood lipids as well.

## Figures and Tables

**Table 1:** The changes in antioxidative defense markers in the liver of C57Bl6 mice treated with nettle extract.

		Experimental Groups			
Antioxidative Defence Markers in Liver		Control Males	Control Females	Nettle Extract Males	Nettle Extract Females
SOD_liver (U/mg protein)	Mean	284.61	163.053	109.998	79.222
	SD	96.597	73.942	23.607	17.101
	Max	614.581	307.435	200.533	144.394
GSH_liver (µmol/mg protein)	Mean	127.666	160.258	119.725 *	96.337 *
	SD	27.069	59.972	20.97	14.227
	Max	292.21	274.222	206.334	194.927
CAT_liver (U/mg protein)	Mean	0.523	0.600	0.487	0.725
	SD	0.112	0.205	0.09	0.114
	Max	1.262	0.955	1.069	1.682

The * marks the statistically significant (*p* ≤ 0.05) differences between the treated groups and the belonging control group of the same sexes by Kruskal–Wallis ANOVA. SOD—activity of the enzyme superoxide dismutase; GSH—the level of the reduced glutathione; CAT—the activity of catalase.

**Table 2:** The changes in blood chemistry liver markers of C57Bl6 mice treated with nettle extract.

			Experimental Groups		
Blood Chemistry Liver Markers		Control Males	Control Females	Nettle Extract Males	Nettle Extract Females
AST (Ul/L)	Mean	161.2	206.0	156.0	189.8
	SD	35.8	13.9	8.0	15.0
	Max	290.0	245.0	110.0	223.0
ALT (U/L)	Mean	65.0	88.7	25.5	137.0
	SD	27.03	59.32	2.06	56.1
	Max	172.0	266.0	30.0	260.0
LDH (U/L) protein)	Mean	706.5	548.0	1024.0 *	1400.0 *
	SD	90.8	88.5	188.9	223.2
	Max	999.0	714.0	1570.0	1895.0

* The asterisk marks the statistically significant (*p* ≤ 0.05) differences between the treated groups and the belonging control group of the same sexes by Kruskal–Wallis ANOVA. SOD—activity of the enzyme superoxide dismutase; GSH—the level of the reduced glutathione; CAT—the activity of catalase.

## Data Availability

The original contributions generated for this study are included in the article; further inquiries can be directed to the corresponding author.

## Abbreviations

TG-total triglycerides, PPAR—peroxisome proliferator activated receptors, LPL—lipoprotein lipase, GLUT—glucose transporters, PGC1-α cofactor of PPAR—gamma, ACOX1—acylCoA oxidase 1, SIRT1—sirtuin (silent mating type information regulation 2 (S. cerevisiae) homolog 1), MDA—malondialdehyde, SOD—superoxide dismutase, CAT—catalase, GSH—reduced glutathione, PBS—phosphate buffer solution, FOXO1—forkhead box protein, Cpt1—palmitoyl transferase, HETE—hydroxyeicosatetraenoic acid family, UCP—uncoupling protein, LXR—liver X receptor (LXR), member of the nuclear receptor family of transcription factors closely related to nuclear receptors PPARs.

## References

[1] Samakar B., Mehri S., Hosseinzadeh H. (2022). A review of the effects of *Urtica dioica* (nettle) in metabolic syndrome. Iran J. Basic Med. Sci..

[2] Mollazadeh H., Mahdian D., Hosseinzadeh H. (2019). Medicinal plants in treatment of hypertriglyceridemia: A review based on their mechanisms and effectiveness. Phytomedicine.

[3] Payab M., Hasani-Ranjbar S., Shahbal N., Qorbani M., Aletaha A., Haghi-Aminjan H., Soltani A., Khatami F., Nikfar S., Hassani S. (2020). Effect of the herbal medicines in obesity and metabolic syndrome: A systematic review and meta-analysis of clinical trials. Phytother. Res..

[4] Ta Tahri A., Yamani S., Legssyer A., Aziz M., Mekhfi H., Bnouham M., Ziyyat A. (2000). Acute diuretic, natriuretic and hypotensive effects of a continuous perfusion of aqueous extract of Urtica dioca in the rat. J. Ethnopharmacol..

[5] Qayyum R., Qamar H.M.U., Khan S., Salma U., Khan T., Shah A.J. (2016). Mechanisms underlying the antihypertensive properties of *Urtica dioica*. J. Transl. Med..

[6] Ghalavand A., Motamedi P., Deleramnasab M., Khodadoust M. (2017). The effect of interval training and nettle supplement on glycemic control and blood pressure in men with type 2 diabetes. Int. J. Basic Sci. Med..

[7] Vajic U.J., Grujic-Milanovic J., Miloradovic Z., Jovovic D., Ivanov M., Karanovic D., Savikin K., Bugarski B., Mihailovic-Stanojevic N. (2018). *Urtica dioica* L. leaf extract modulates blood pressure and oxidative stress in spontaneously hypertensive rats. Phytomedicine.

[8] Petlevski R., Hadžija M., Slijepčević M., Juretić D. (2001). Effect of ‘antidiabetis’ herbal preparation on serum glucose and fructosamine in NOD mice. J. Ethnopharmacol..

[9] Golalipour M.J., Ghafari S., Kouri V., Kestkar A.A. (2010). Proliferation of the β -cells of pancreas in diabetic rats treated with *Urtica dioica*. Int. J. Morphol..

[10] Sahraki M.R., Mirshekari H., Sahraki A.R., Shafighi E. (2013). Effect of *Urtica dioica* decoction on serum glucose and lipid profile in streptozotocin induced diabetic male rats. Zahedan J. Res. Med. Sci..

[11] Dabagh S., Nikbakht M. (2016). Glycemic control by exercise and *Urtica dioica* Supplements in men with type 2 diabetes. Jundishapur J. Chronic Disease. Care.

[12] Khalili N., Fereydoonzadeh R., Mohtashami R., Mehrzadi S., Heydari M., Huseini H.F. (2017). Silymarin, olibanum, and nettle, a mixed herbal formulation in the treatment of type II diabetes: A randomized, double-Blind, placebo-controlled, clinical trial. Evid. Based Complement. Alternat. Med..

[13] Gohari A., Noorafshan A., Akmali M., Zamani-Garmsiri F., Seghatoleslam A. (2018). *Urtica dioica* distillate regenerates pancreatic beta cells in streptozotocin-induced diabetic rats. Iran J. Med. Sci..

[14] Khare V., Kushwaha P., Verma S., Gupta A., Srivastava S., Rawat A. (2012). Pharmacognostic evaluation and antioxidant activity of *Urtica dioica* L.. Chin. Med..

[15] Hanczakowska E., Świątkiewicz M., Grela E.R. (2015). Effect of dietary inclusion of a herbal extract mixture and different oils on pig performance and meat quality. Meat Sci..

[16] Musunuru K. (2010). Atherogenic dyslipidemia: Cardiovascular risk and dietary intervention. Lipids.

[17] Mahjoub S., Davari S., Moazezi Z., Qujeq D. (2012). Hypolipidemic effects of ethanolic and aqueous extracts of *Urtica dioica* in rats. World Appl. Sci. J..

[18] Vengerovsky A.I., Yakimova T.V., Nasanova O.N. (2015). The influence of nettle and burdock extracts in combination with different diets on dyslipidemia in diabetes mellitus model. Vopr. Pitan..

[19] Dar S.A., Ganai F.A., Yousuf A.R., Balkhi M.U.H., Bhat T.M., Sharma P. (2013). Pharmacological and toxicological evaluation of *Urtica dioica*. Pharm. Biol..

[20] Raimova K., Abdulladjanova N., Kurbanova M., Makhmanov D., Kadirova Sh O., Tashpulatov F. (2020). Comprehensive study of the chemical composition of *Urtica dioica* L.. J. Crit. Rev..

[21] Jeong S.M., Kang M.J., Choi H.N., Kim J.H., Kim J.I. (2012). Quercetin ameliorates hyperglycemia and dyslipidemia and improves antioxidant status in type 2 diabetic db/db mice. Nutr. Res. Prac..

[22] Madadi Jaberi M., Vahidian Rezazadeh M., Mogharnasi M., Karaji Bani M. (2016). The effect of 8 weeks of aerobic training and consumption of hydro-alcoholic extract of nettle on apelin and hs-CRP plasma levels of overweight and obese women. Armaghane Danesh..

[23] Abedinzade M., Rostampour M., Mirzajani E., Khalesi Z.B., Pourmirzaee T., Khanaki K. (2019). *Urtica dioica* and *Lamium album* decrease glycogen synthase kinase-3 beta and increase K-Ras in diabetic rats. J. Pharmacopunct..

[24] Rau O., Wurglics M., Dingermann T., Abdel-Tawab M., Schubert-Zsilavecz M. (2006). Screening of herbal extracts for activation of the human peroxisome proliferator-activated receptor. Pharmazie.

[25] Fan S., Raychaudhuri S., Kraus O., Shahinozzaman M., Lofti L., Obanda D.N. (2020). *Urtica dioica* Whole Vegetable as a Functional Food Targeting Fat Accumulation and Insulin Resistance-a Preliminary Study in a Mouse Pre-Diabetic Model. Nutrients.

[26] Committee for the Update of the Guide for the Care and Use of Laboratory Animals (2011). Guide for the Care and Use of Laboratory Animals.

[27] Elez Garofulić I., Malin V., Repajić M., Zorić Z., Pedisić S., Sterniša M., Smole Možina S., Dragović-Uzelac V. (2021). Phenolic Profile, Antioxidant Capacity and Antimicrobial Activity of Nettle Leaves Extracts Obtained by Advanced Extraction Techniques. Molecules.

[28] Nair A.B., Jacob S. (2016). A simple practice guide for dose conversion between animals and human. J. Basic Clin. Pharm..

[29] Friedewald W.T., Levy R.I., Fredrickson D.S. (1972). Estimation of the concentration of low-density lipoprotein cholesterol in plasma, without use of the preparative ultracentrifuge. Clin. Chem..

[30] Oršolić N., Landeka Jurčević I., Đikić D., Rogić D., Odeh D., Balta V., Perak Junaković E., Terzić S., Jutrić D. (2019). Effect of propolis on diet-induced hyperlipidemia and atherogenic indices in mice. Antioxidants.

[31] Lowry O.H., Rosebrough N.J., Farr A.L., Randall R.J. (1951). Protein measurement with the Folin phenol reagent. J. Biol. Chem..

[32] Landeka Jurčević I., Dora M., Guberović I., Petras M., Rimac S., Brnčić S., Đikić D. (2017). Polyphenols from wine lees as a novel functional bioactive compound in the protection against oxidative stress and hyperlipidaemia. Food Technol. Biotechnol..

[33] Colombo G., Clerici M., Garavaglia M.E., Giustarini D., Rossi R., Milzani A., Dalle-Donne I. (2016). A step-by-step protocol for assaying protein carbonylation in biological samples. J. Chromatogr. B Analyt. Technol. Biomed. Life Sci..

[34] GraphPad Software. https://www.graphpad.com/.

[35] Dadvar N., Ghalavand A., Zakerkish M., Hojat S., Alijani E., Mahmoodkhani Kooshkaki R. (2016). The effect of aerobic training and *Urtica dioica* on lipid profile and fasting blood glucose in middle age female with type II diabetes. Jundishapur. Sci. Med. J..

[36] Chatterji S., Fogel D. (2018). Study of the effect of the herbal composition SR2004 on hemoglobin A1c, fasting blood glucose, and lipids in patients with type 2 diabetes mellitus. Integr. Med. Res..

[37] Mehran M.M., Norasfard M.R., Abedinzade M., Khanaki K. (2015). *Lamium album* or *Urtica dioica*? which is more effective in decreasing serum glucose, lipid and hepatic enzymes in streptozotocin induced diabetic rats: A comparative study. Afr. J. Tradit. Complement. Altern. Med..

[38] Das M., Sarma B., Rokeya B., Parial R., Nahar N., Mosihuzzaman M., Khan A., Ali L. (2011). Antihyperglycemic and antihyperlipidemic activity of *Urtica dioica* on type 2 diabetic model rats. J. Diabetol..

[39] Nassiri-Asl M., Zamansoltani F., Abbasi E., Daneshi M.-M., Zangivand A.-A. (1986). Effects of *Urtica dioica* extract on lipid profile in hypercholesterolemic rats. J. Chin. Integr. Med..

[40] Kotoh K., Kato M., Kohjima M., Tanaka M., Miyazaki M., Nakamura K., Enjoji M., Nakamuta M., Takayanagi R. (2011). Lactate dehydrogenase production in hepatocytes is increased at an early stage of acute liver failure. Exp. Ther. Med..

[41] Pourahmadi M., Jashni H.K., Bagheri M., Sotoodeh Jahromi A. (2014). The effect of hydro-alcoholic extract of *Urtica dioica* Root on testes in adult rats. Life Sci. J..

[42] Grabacka M., Pierzchalska M., Dean M., Reiss K. (2016). Regulation of Ketone Body Metabolism and the Role of PPARα. Int. J. Mol. Sci..

[43] Kersten S. (2014). Integrated physiology and systems biology of PPARα. Mol. Metab..

[44] Ahmadian M., Suh J.M., Hah N., Liddle C., Atkins A.R., Downes M., Evans R.M. (2013). PPARγ signaling and metabolism: The good, the bad and the future. Nat. Med..

[45] Rodgers J.T., Lerin C., Haas W., Gygi S.P., Spiegelman B.M., Puigserver P. (2005). Nutrient control of glucose homeostasis through a complex of PGC-1-α and SIRT1. Nature.

[46] McGinnis C.D., Jennings E.Q., Harris P.S., Galligan J.J., Fritz K.S. (2022). Biochemical Mechanisms of Sirtuin-Directed Protein Acylation in Hepatic Pathologies of Mitochondrial Dysfunction. Cells.

[47] Souza S.A., Held A., Lu W.J., Drouhard B., Avila B., Leyva-Montes R., Hu M., Miller B.R., Ng H.L. (2021). Mechanisms of allosteric and mixed mode aromatase inhibitors. RSC Chem. Biol..

